# P-106. Preliminary Results of the Comparing Oral versus Parenteral Antimicrobial Therapy (COPAT) Clinical Trial

**DOI:** 10.1093/ofid/ofae631.313

**Published:** 2025-01-29

**Authors:** Joy J Juskowich, Jesse M Thompson, John A Guilfoose, Allison Lastinger, Jonathan E Stanley, Connie L Smith, Victor A Arcega, Seyoum Bage, Arif R Sarwari

**Affiliations:** West Virginia University, Morgantown, West Virginia; West Virginia University, Morgantown, West Virginia; West Virginia University, Morgantown, West Virginia; West Virginia University Hospital, Morgantown, West Virginia; WVU Medicine, Bridgeport, West Virginia; West Virginia University, Morgantown, West Virginia; West Virginia University, Morgantown, West Virginia; WVU Medicine, Bridgeport, West Virginia; West Virginia University, Morgantown, West Virginia

## Abstract

**Background:**

Early intravenous (IV) to oral (PO) antibiotic transition for serious infections such as bacteremia, endocarditis, and bone/joint infections is noninferior to continued IV antibiotics. Our experience shows that Complex Outpatient Antimicrobial Therapy with PO antimicrobials (COpAT) is not only clinically effective but much safer. However, COpAT programs are not yet widely incorporated into clinical practice. **C**omparing **O**ral vs. **P**arenteral **A**ntimicrobial **T**herapy (COPAT) Trial was designed to demonstrate improved safety with equivalent effectiveness of early IV to PO antibiotic transition across almost all infectious diseases. We hypothesized that, without compromising clinical outcomes, adverse events (readmissions, antibiotic toxicities, and line issues) will be significantly lower in patients discharged with PO antibiotics.

**Figure:**
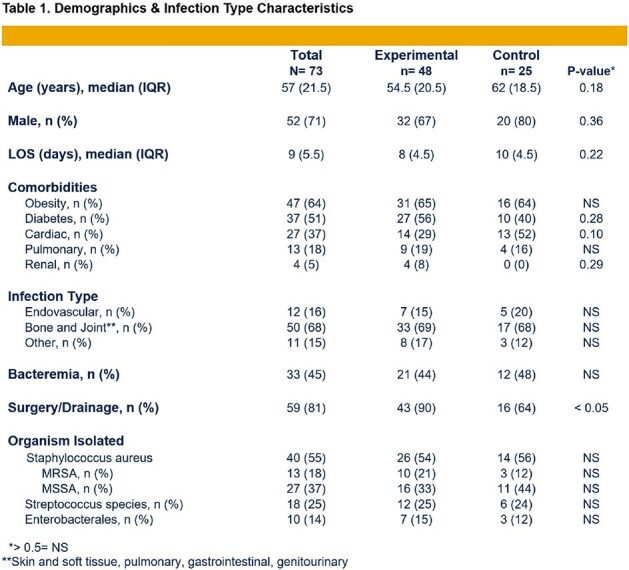

**Methods:**

COPAT Trial is an ongoing open-label, pragmatic, randomized controlled trial at five Infectious Disease faculty staffed hospitals in one health system. A total 135 hospitalized patients otherwise being discharged with two or more weeks of IV antibiotics are randomized 2:1 to early PO (*Experimental*) vs. continued IV (*Control*) antibiotics. Two primary outcomes, assessed at 12 weeks, are efficacy equivalence and *Experimental* group safety superiority. From Aug 4, 2023 through Apr 30, 2024, 73 (*Experimental* 48, *Control* 25) active patients were enrolled and 64 (*Experimental* 42, *Control* 22) completed the trial.

**Figure:**
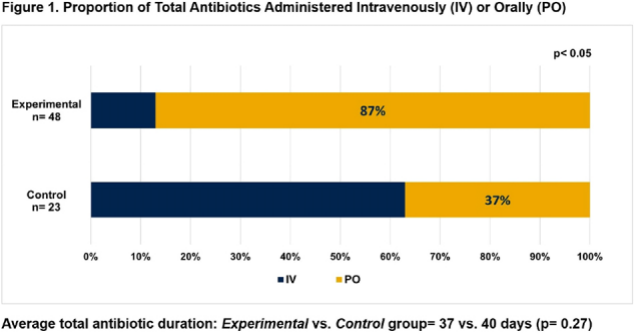

**Results:**

Demographics and infection type were similarly distributed between both groups with a predominance of bone/joint followed by endovascular infections (Table 1). While total antibiotic durations were comparable, *Experimental* group received disproportionately more PO antibiotics (87 vs. 37%, p< 0.05) (Figure 1). Both groups had a strong preference for PO antibiotics, and all patients felt their infection had been successfully treated (Table 2). *Experimental* group had a significantly lower total adverse event rate (2.8 vs. 7.2%, p< 0.05) (Table 3). All patients achieved clinical cure at 12 weeks.

**Figure:**
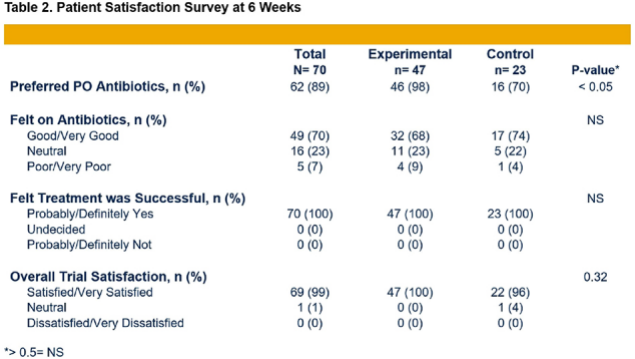

**Conclusion:**

Early IV to PO transition significantly improves antimicrobial treatment safety without compromising effectiveness across many serious infectious diseases. Patients report a strong preference for PO antibiotics.

**Figure:**
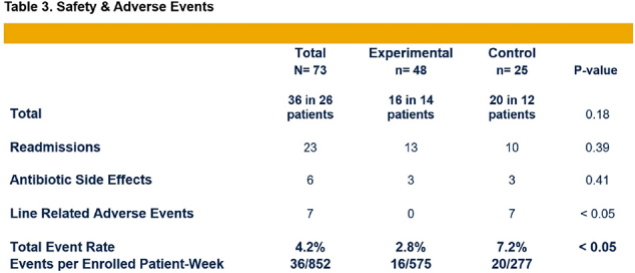

**Disclosures:**

**All Authors**: No reported disclosures

